# Quality of Life in People With Unilateral Lower Limb Amputation at a Tertiary Rehabilitation Centre in Northern India: A Cross-Sectional Study

**DOI:** 10.7759/cureus.36985

**Published:** 2023-03-31

**Authors:** Deepak K, Dileep Kumar, Sudhir R Mishra, Anil K Gupta, Ganesh Yadav

**Affiliations:** 1 Physical Medicine and Rehabilitation, King George's Medical University, Lucknow, IND

**Keywords:** transfemoral amputation, transtibial amputation, rta-road traffic accidents, quality of life (whoqol-bref questionnaire), quality of life (qol)

## Abstract

Background and purpose

The patients after amputation undergo a sudden transformation in their quality of life. In India, amputation done at the appropriate time is a rare phenomenon because usually, the patients present themselves at the later stages. The surgeons, however, while performing amputation surgeries, primarily consider saving the life of a patient under adverse conditions when patients report to them very late that the surgeries are carried out urgently. Assessing the quality of life (QOL) and the various sociodemographic factors affecting the QOL paves the way for future rehabilitation programs.

Aims and objectives

To evaluate the quality of life of subjects with unilateral lower limb amputation among the North Indian population.

Materials and methods

This cross-sectional study was conducted in the tertiary rehabilitation center. A total of 106 Subjects were recruited. Informed consent was taken. WHOQOL-BREF contains 26 items covering four important aspects of QOL. The WHOQOL-BREF self-administered free questionnaire was used as a data collection tool, and the Hindi version downloaded from the WHO website was also used for those who can't understand English.

Results

The range of the physical domain, psychological domain, social domain, and environmental domain were 0 and 100. The mean score of different QOL transformed domain scores (on a scale of 100) were 47.91±20.12, 57.37±20.46, 59.36±25.32 and 51.50±21.96, respectively. Trauma was the leading cause of amputation, followed by diabetes mellitus, cancer, peripheral vascular disease, and other causes. Transtibial amputees were more in number compared to transfemoral. The percentage of male and female amputees was 78.30%, and 21.70%, respectively.

Conclusion

The physical domain was the most affected domain, followed by the psychological, social, and environmental domains. A delay in the prosthesis fitment aggravates the physical burden of amputees. Early prosthesis & psychological counseling will improve the QOL significantly.

## Introduction

Amputation is the surgical removal of a limb or body part. It is performed to remove diseased tissue or to relieve pain or/due to trauma. It is done when the salvage of the limb is not possible. Lower limb amputation is one of the oldest surgical procedures in the history of surgery [1]. Trauma is the ultimate cause which alone leads to 16 percent of the total burden of all the diseases in the world. In our country, RTA is the most common cause of mortality and morbidity. Amputation causes permanent disfiguration and disability. The mobility of the people is affected to a larger extent [2]. It also makes the individuals dependent on others. It also affects people in their social, economic, and psychological aspects.

World Health Organization (WHO) defines the quality of life as an individual’s perception of their position in life in the context of the culture and value systems in which they live and in relation to their goals, expectations, standards, and concern [1,3]. WHO framed a free questionnaire for assessing the quality of life of the people, which is known as the WHOQOL-BREF questionnaire. WHOQOL-BREF evaluates the quality of life in four different aspects, i.e., physical health, psychological, social relationship, and environmental health [4].

The main function of the lower limb is the locomotion function. Once the locomotion function is affected, it will interfere with the employment of the patient. It also affects the economic, social, and psychological impact of the patient as well as his family members [5]. Therefore, it is very important to assess the quality of life of these people with lower limb amputation. There is a lack of information on the quality of life in people living with lower limb amputation in developing countries like India.

In North India, there is no such study to the best of our knowledge to describe the information about the quality of life in unilateral lower limb amputees. The main aim was to assess the various sociodemographic factors affecting the QOL of the lower limb amputees. The future holistic approach to rehabilitation programs for people with lower limb amputation can be improved by this study.

## Materials and methods

This is a cross-sectional study. Subjects with unilateral lower limb amputees were recruited from indoor and Outdoor facilities of the Department of Physical Medicine & Rehabilitation or referred from any other department. Informed consent was taken. The WHOQOL-BREF self-administered questionnaire was used as a data collection tool. We conducted this study on the North Indian population. For those who can’t understand the English version of WHOQOL-BREF, we used the Hindi version of the WHOQOL-BREF questionnaire.

The study was approved by IRB/Committee: King George's Medical University Institutional Ethics Committee (Approval #: 107th ECM II B- Thesis/P3 dated 22/04/2021).

Sample size and statistical analysis

A sample size of 106 was obtained with 95% confidence level and 90% power of a similar study [2]. Significance was assessed at a 5% level of significance. The results were analyzed using IBM Corp. Released 2013. IBM SPSS Statistics for Windows, Version 22.0. Armonk, NY: IBM Corp.

Inclusion criteria

1) Age: 18 years and above, 2) patients with unilateral lower limb amputation, 3) post-amputation with duration over three months, 4) subjects willing to participate and to give informed consent.

Exclusion criteria

1) Patients with upper limb and/or bilateral lower limb amputation, 2) post-amputation with a duration less than three months, 3) subjects who refused to participate, 4) patients with hearing, speech, and visual function disorders and other associated polytrauma like TBI, SCI, etc., 5) patients with psychiatric illness.

## Results

The percentage of <20 years, 20-39 years, 40-59 years, and >60 years age groups were 4.72%, 48.11%, 32.08%, 15.09%, and 100%, respectively. The majority of the patients belonged to the 20 - 39 years old age group, as shown in Table 1.

**Table 1:** Distribution of patients according to different age groups

Age	n	%
<20 years	5	4.72
20-39 years	51	48.11
40-59 years	34	32.08
>60 years	16	15.09
Total	106	100

The percentage of males and females was 78.30%, and 21.70%, respectively. The most common cause of amputation was road traffic accidents, as shown in Table 2.

**Table 2:** Distribution of patients according to causes of amputation

Causes of amputation	n	%
Road Traffic Accident	73	68.87
Diabetes Mellitus	9	8.49
Cancer	9	8.49
Peripheral Vascular Disease	8	7.55
Other causes	7	6.60

The percentage of transtibial amputation, transfemoral amputation, and others were 63.21%, 34.91%, and 1.89%, respectively. The percentage of illiterate, primary school, middle school, high school, and beyond high school was 23.58%, 9.43%, 16.98%, 20.75%, and 29.25%, respectively. The percentage of working, pension/service, unemployment, and dependent was 26.42%, 6.60%, 4.72%, and 62.26%, respectively. Most amputees lost their job after amputation. The duration of prosthesis use is shown in Table 3.

**Table 3:** Distribution of patients according to the duration of prosthesis use

Duration of prosthesis use	numbers	%
No prosthesis	65	61.32
<6 months	2	1.89
6-12 months	2	1.89
1-5 years	5	4.72
>5 years	31	29.25

The range of the physical domain, psychological domain, social domain, and environmental domain were 0 and 100. The mean score of different QOL transformed domain scores (on a scale of 100) were 47.91±20.12, 57.37±20.46, 59.36±25.32 and 51.50±21.96, respectively. The physical domain has the least score among all, as shown in Table 4.

**Table 4:** The mean score of different QOL transformed domain scores (on a scale of 100) among study participants

	Mean	Median	Std. Deviation	Minimum	Maximum	Percentiles	
						25	75
Physical Domain	47.91	38.00	20.12	0	100	31	63
Psychological Domain	57.37	56.00	20.46	0	100	44	69
Social Domain	59.36	69.00	25.32	0	100	44	75
Environmental Domain	51.50	50.00	21.96	0	100	38	63

The mean physical domain and environment domain scores were significantly different between different educational statuses. Moreover, the mean psychological domain and social domain were not significantly different between different Educational statuses, as shown in Table 5.

**Table 5:** Association of educational status with QOL score within the total physical domain, psychological domain, social domain, and environmental domain

Educational status	Physical domain		Psychological Domain		Social Domain		Environment Domain	
	Mean	±SD	Mean	±SD	Mean	±SD	Mean	±SD
Illiterate	17.84	4.50	17.72	4.45	9.36	3.00	21.16	5.60
Primary school	17.00	5.08	19.90	4.36	9.80	2.49	21.20	4.92
Middle school	18.67	5.10	19.22	5.06	10.39	2.70	23.50	7.64
High school	21.09	5.34	19.59	4.97	9.32	3.14	24.50	7.99
Beyond High school	23.29	6.02	21.68	4.87	11.10	3.14	27.06	6.58
p-Value	0.001^*^		0.051		0.158		0.016^*^	

The mean physical domain, psychological domain, and environmental domain scores were significantly different between different age groups, as shown in Table 6.

**Table 6:** Association of different age groups with QOL score

Age group	Physical domain		Psychological Domain		Social Domain		Environment Domain	
	Mean	±SD	Mean	±SD	Mean	±SD	Mean	±SD
<20 years	25.80	5.17	26.60	1.14	11.80	1.10	32.60	4.72
20-39 years	20.51	6.03	19.65	5.13	10.14	3.35	23.25	7.78
40-59 years	18.59	5.12	18.41	4.25	9.38	2.73	22.65	5.27
>60 years	20.69	5.13	20.63	4.54	10.81	2.69	26.44	6.31
p-Value	0.049^*^		0.004^*^		0.224		0.009^*^	

The mean physical domain, psychological domain, and environmental domain scores were significantly different between different financial statuses. Moreover, the mean social domain was not significantly different between different financial statuses, as shown in Table 7.

**Table 7:** Association of financial status with QOL score within the total physical domain, psychological domain, social domain, and environmental domain

Financial status Income (in rupees)	Physical Domain		Psychological Domain		Social Domain		Environmental Domain	
	Mean	±SD	Mean	±SD	Mean	±SD	Mean	±SD
<5000/-	18.10	4.47	18.93	4.26	9.81	2.68	22.52	5.30
5000-10000/-	20.09	5.69	19.18	5.30	9.64	2.94	22.82	7.87
10000-20000/-	21.18	6.01	18.76	5.31	9.29	3.06	23.00	8.35
>20000/-	23.04	6.33	22.20	4.78	11.44	3.36	28.12	6.62
p-Value	0.005^*^		0.037^*^		0.069		0.008^*^	

## Discussion

Factors affecting QOL

Age

The mean physical domain, psychological domain, and environmental domain scores were significantly different in different age groups. G.D. Pooja et al., in 2012, did a study in Kolkata and also found that the majority of amputees who were of working age lost their jobs due to their decreased physical capacity [6].

Male vs. Female

Several studies showed a higher prevalence of lower limb amputation in males compared to females. Sinha et al. did a study in Mumbai and also found that most of the amputees were male, comprising 88% [1]. A study done by Hawkins et al. in 2016 quotes the males female ratio as 4:1 [1]. The preponderance of male amputees compared with female amputees is the fact that it is that the male community moves outside in search of job opportunities [7].

Education and QOL

The mean physical domain and environment domain scores were significantly different between different educational statuses. People with a good education have better adaptability to post-amputation body changes and have good exposure to existing healthcare services.

Work and QOL

In the current study, the mean physical domain and environmental domain scores were significantly different between different educational statuses. Many amputees, who were only the earning members of their families, lost their job and became totally dependent. Grewal VS et al. did a study in 2019 that also stated that employment status is an important determinant of QOL [2].

Income and QOL

In the current study, the mean physical domain, psychological domain, and environmental domain scores were significantly different between different financial statuses. Grewal VS et al. did a study in 2019 that also states that the overall QOL score was highest for the study participants belonging to the upper socio-economic class [1,2]. People with better financial stability ensure access to high-quality curative and rehabilitative services, carefree access to essential items of daily living, both for self and family, and a sense of general well-being factors that have a direct impact on an individual's perception of QOL [1].

Socio-demographic factors

Causes of Amputation

The percentage of RTA, DM, cancer, PVD, and other causes of amputation were 68.87%, 8.49%, 8.49%, 7.55%, and 6.60%, respectively. O’Keeffe and Rout did a study in 2019 and also reported that the most common cause of amputation was trauma [8]. Pooja and Sangeeta did a study in Kolkata and also reported that trauma alone accounts for 70.3 percent of amputation, except in the age group > 60 years [6]. In 2008, Raichle et al. reported trauma was the most common reason for amputations (53.5%), followed by infection (23.4%), peripheral vascular disease (22.3%), and gangrene (20.9%) [9].

According to A. Esquenazi causes of amputations may vary from country to country, but in developing counties, trauma alone accounts for more than 80%. In developed nations like the US and Japan, the vascular disease leads to the major cause of amputation [10].

Level of Amputation

The percentage of transtibial amputation, transfemoral amputation, and others were 63.21%, 34.91%, and 1.89%, respectively. Unnikrishnan et al. did a study in 2017 and also found transtibial amputation was more than 53.1%, while transfemoral amputation was 37.1% [11]. Raichle et al. did a study and also found transtibial amputation (56.1%) was more common, followed by TFA (30.1%). Hawkins et al. reported 78% of lower limb amputations were transtibial amputations [7]. G Singh et al. also found that transtibial amputation was common (67.78%) [12].

Prosthesis vs. Nonprosthesis User

The percentage of people using and without prostheses was 39.62% and 60.38%, respectively. Prosthesis provides support for mobility in the workplace. Amputees who didn't receive their prostheses can't resume their work [13-17]. Due to decreased physical capacity, most amputees need to change to less physically demanding jobs [13-17]. 

Stump-Related Problems

The percentage of people with and without stump-related problems was 20.75% and 79.25%, respectively. Stump-related problems like phantom limb pain, residual limb pain, reduced range of motion, ulcers, discharging sinus, and bony prominence, etc., were the main reasons for the delay in being fitted with a prosthesis [17]. Lack of early rehabilitation has a direct impact on stump-related problems. Most of the amputees with stump issues required revision of amputation surgery.

Limitations of the study

Direct analysis of the amputee's functional competence was not done. Selection bias could have influenced our results because only subjects visiting our hospital were enrolled.

## Conclusions

The physical domain was the most affected of all, followed by the psychological domain. Stump-related problems, financial burdens, and delayed rehabilitation lead to delayed prosthesis fitment. This ultimately paved the way for their decreased physical capacity. These points should be kept in mind for future rehabilitation purposes.
